# Moving beyond quantity of participation in process evaluation of an intervention to prevent excessive pregnancy weight gain

**DOI:** 10.1186/1479-5868-10-23

**Published:** 2013-02-13

**Authors:** Keriann H Paul, Christine M Olson

**Affiliations:** 1 Division of Nutritional Sciences, Cornell University, 406 Savage Hall, Ithaca, NY, 14853, USA

**Keywords:** Pregnancy, Weight gain, Goal setting, Participation, Process evaluation

## Abstract

**Background:**

Few lifestyle interventions have successfully prevented excessive gestational weight gain. Understanding the program processes through which successful interventions achieve outcomes is important for the design of effective programs. The objective of this study was to evaluate the effect of the quantity and quality of participation in a healthy lifestyle intervention on risk of excessive gestational weight gain.

**Findings:**

Pregnant women (N = 179) received five newsletters about weight, nutrition, and exercise plus postcards on which they were asked to set related goals and return to investigators. The quantity of participation (dose) was defined as low for returning few or some vs. high for many postcards (N = 89, 49.7%). Quality of participation was low for setting few vs. high for some or many appropriate goals (N = 92, 51.4%). Fisher’s exact tests and multivariate logistic regression were used to analyze the effect of participation variables on the proportion with excessive weight gain. Quantity and quality of participation alone were each not significantly associated with excessive gestational weight gain, while quality of participation among those with high-levels of participation approached significance (p = 0.07). The odds of gaining excessively was decreased when women had both a high quantity and quality of participation (OR = 0.04, 95% CI = 0.005, 0.30).

**Conclusions:**

Both quantity and quality of participation are important program process measures in evaluations of lifestyle interventions to promote healthy weight gain during pregnancy.

## Introduction

Avoiding excessive gestational weight gain, defined as gaining above the Institute of Medicine’s (IOM) gestational weight gain (GWG) recommendations, has become a priority in obesity prevention [1]. Excessive GWG is a risk factor for postpartum weight retention that contributes to long-term weight gain [1]. The prevalence of excessive GWG is high. In the US, 38.4% of normal weight women and 63% and 46.3% of overweight and obese women, respectively, gain more than recommended [1].

Systematic reviews and meta-analyses have examined the effects of interventions on risk of excessive weight gain during pregnancy [2-6]. The results are mixed, but generally indicate a positive effect on reduced risk. The data for the study reported here come from a study that is included in three of these reviews [2-4,7]. In their review, Gardner, Wardle and Croker [2] call for the inclusion of more information on the intervention design, content, and delivery as a way to explain the variations in the effectiveness of trials.

Process evaluation aids understanding of how interventions achieve their intended outcomes. A key process measure is dose of intervention received. For interventions around weight control, diet, and physical activity, amount of participation (e.g. the number of group sessions attended) is often used as a measure of dose received. A higher dose of intervention received has generally been associated with greater success in achieving intervention outcomes [8-10].

The objective of this study was to use data on program participation from a previously published trial to evaluate the impact of the quantity and quality of participation in a newsletter-based healthy lifestyle intervention for pregnant women on risk of excessive GWG.

## Methods

The Bassett Mothers Health Project *too* (BMHP II), the data source for the present study, found that total gestational weight gain did not differ significantly between the historical control group that received standard care and the intervention group (14.8 ± 4.68 kg; N = 381 vs. 14.10 ± 4.51 kg; N = 179; p = .09). However, among low-income women, there was a significant difference between control and intervention groups in the proportion with excessive GWG (52% vs. 33%; p < .01) [7]. The 1990 IOM recommendations were the basis for determining excessive GWG since these were the guidelines available at the time of the study and those around which the intervention was designed [11]. Additional information on the design, methods and results are available in the original publication of the efficacy trial [7].

In BMHP II, women in the intervention group were mailed five newsletters on weight gain, diet, and physical activity between weeks 14 and 32 of their pregnancies. The timing of the mailings varied by time of entry into the study which could have been from the beginning of pregnancy up to the 28^th^ week of pregnancy. Each newsletter included a postcard that prompted women to set a specific goal related to the newsletter topic and return it by mail to investigators. The BMHP II used postcards returned to assess participation in the intervention [7]. The quantity of participation was measured by the number of postcards returned. The goal was for women to return 4 postcards. For the quality of participation, the appropriateness of goals was evaluated and then the number of appropriate goals served as the indicator for quality of participation. A target number of high quality goals was not determined before the study. Goals were considered as appropriate if they were positive vs. negative and specific vs. vague. For quantity of participation, women were categorized as follows: Few or some = 0–3 postcards returned (Low) vs. many = 4–5 postcards returned (High). For quality, women were categorized as few = 0–1 appropriate goals (Low) vs. some or many = 2–4 appropriate goals (High). The physical activity goal was excluded from the quality of participation variable because it was structured so that it was automatically appropriate, if it was filled in on the postcard. The cut-offs were selected to optimize the distribution of data across categories.

Within the intervention group of 179 women, the participation measures were initially compared to the proportion gaining above the range using Fisher’s exact tests. Multiple logistic regression analysis was used to evaluate the effect of the two participation measures on the odds of gaining above the range while adjusting for covariates for age, parity, income, cigarette smoking, food intake, body weight category, significant weight loss in early pregnancy, and timing of measurements, as previously described [7]. P-values < .05 were considered statistically significant. All analyses were conducted using SAS software (version 9.2, SAS Institute, Cary, NC).

## Findings

### Participation and gestational weight gain

In the intervention group women, quantity of participation (number of postcards returned) was not significantly associated with the proportion gaining above the recommended GWG range. The percentage of women who gained above the range was actually higher among women with a high quantity of participation compared to those with lower participation (42.7% vs. 38.9%), but not statistically significant. Quality of participation was also not significantly associated with excessive gestational weight gain (41.3% vs. 40.0%). Within the sub-group of 89 women with a high quantity of participation, women with high quality participation were less likely to gain above the range compared to those with lower quality participation. This difference approached significance (38.2% vs. 61.8% p = 0.07). No association was seen in the group of 90 women with a low quantity of participation.

The interaction between the quantity and quality of participation was significant in a multiple logistic regression model that adjusted for covariates and timing of measurement variables (p = 0.002; OR = 0.04, 95% CI = 0.005, 0.30). A high quantity of participation and a high quality of participation significantly decreased the odds of gaining above the range compared to having a high quantity of participation and low quality of participation (OR = 0.15, 95% CI = 0.03, 0.70). Being overweight and eating much more food during pregnancy significantly increased the odds of gaining above the range, OR = 4.75, 95% CI = 2.0, 11.3 and OR = 5.22, 95% CI = 1.9, 14.4, respectively. The results for these covariates replicate previous findings [12].

## Discussion

A high dose or quantity of participation was not associated with a decreased risk of gaining above the recommended weight gain range. Among women with a high amount of participation, defined as returning 4 or more postcards, a high quality participation (setting 2 or more appropriate goals) was significantly related to decreased risk of excessive gestational weight gain.

Generally, participation measured as attendance (i.e. dose received), is associated with greater success in face-to-face weight management interventions [8-10]. The relationship between dose received and successful outcomes for community-based interventions is not as strong. The Pound of Prevention pilot study in Minnesota similarly used postcards to monitor participation with the weight maintenance intervention and found the number of postcards returned was only marginally associated with weight loss [13]. A correspondence-based postpartum weight loss intervention found that the number of returned homework assignments, a basic measure of participation, was not associated with success in the intervention [14]. The number of self-monitoring records, however, was significantly correlated to weight loss. Self-monitoring has recently been identified as an effective technique to improve physical activity and healthy eating, and interventions that included self-monitoring and another component of control theory, goal setting, were even more effective [15]. Similarly, full-participation in goal setting activities in a work-site intervention to improve physical activity was not related to changes in physical activity, but there was a dose response relationship between the difficulty of goals and change in physical activity [16,17]. Thus, the quality of participation seems to be important for success in non-face-to-face interventions and should be included in process evaluations along with dose received.

Goal setting is a complex process that includes: 1) recognizing the need for change via some type of assessment, 2) establishing a goal, 3) committing to the goal, 4) monitoring goal-related activities, and 5) self-rewarding for goal attainment [18]. Ideally, goals should be difficult yet attainable, specific and measureable, and set for a specific period of time [18]. Although goal setting is generally found to be an effective dietary and physical activity behavior change strategy, few nutrition and physical activity interventions actually implement the full goal setting process. Consequently, few have explicitly examined the quality of goals set by participants as part of process evaluation [18]. Our instructions on the postcards were to write down, “one specific change,” related to the topic of the newsletter. There were elements of assessment and self-monitoring in some of the newsletters and postcards. Thus goal setting for this intervention was mostly confined to step 2, establishing a goal, so only the qualitative aspects of this component were examined.

Contemporary non-face-to-face interventions are increasingly web-based and participation (e.g. viewing materials) can be easily monitored by tracking website visits. A web-based program with online goal-setting to improve self-management of epilepsy found that although participation was high (many goals set), 68% of goals were too general or only somewhat specific [19]. Despite having offered guidelines for writing goals, the authors concluded that participants needed more assistance with goal-setting in order to facilitate behavior change. Goal-setting interventions with no face-to-face interaction need to provide support for setting quality goals either through detailed instructions or through a guided-goal setting tool [18,20,21]. Finally, qualitative research could help to identify both important program processes from the participants’ perspectives and alternative ways of assessing both the quantity and quality of participation with community-based interventions [22].

## Abbreviations

OR: Odds ratio; CI: Confidence interval; IOM: Institute of Medicine; GWG: Gestational weight gain; BMHP II: Bassett Mothers Health Project *too!*; US: United States; SAS: Statistical Analysis System.

## Competing interests

The authors declare that they have no competing interests.

## Authors’ contributions

CMO led the original study, CMO and KHP conceptualized the research presented in the manuscript and jointly wrote the manuscript, and KHP conducted the data analysis. Both authors have approved the final manuscript.
